# Brain aging differs with cognitive ability regardless of education

**DOI:** 10.1038/s41598-022-17727-6

**Published:** 2022-08-16

**Authors:** Kristine B. Walhovd, Lars Nyberg, Ulman Lindenberger, Inge K. Amlien, Øystein Sørensen, Yunpeng Wang, Athanasia M. Mowinckel, Rogier A. Kievit, Klaus P. Ebmeier, David Bartrés-Faz, Simone Kühn, Carl-Johan Boraxbekk, Paolo Ghisletta, Kathrine Skak Madsen, Willliam F. C. Baaré, Enikő Zsoldos, Fredrik Magnussen, Didac Vidal-Piñeiro, Brenda Penninx, Anders M. Fjell

**Affiliations:** 1 Center for Lifespan Changes in Brain and Cognition, University of Oslo, Blindern, POB1094, 0317 Oslo, Norway; 2 Department of Radiology and Nuclear Medicine, Oslo University Hospital, Oslo, Norway; 3 Umeå Center for Functional Brain Imaging, Umeå University, Umeå, Sweden; 4 Center for Lifespan Psychology, Max Planck Institute for Human Development, Berlin, Germany; 5 Max Planck UCL Centre for Computational Psychiatry and Ageing Research, Berlin, Germany; 6 Max Planck UCL Centre for Computational Psychiatry and Ageing Research, London, UK; 7 Cognitive Neuroscience Department, Donders Institute for Brain, Cognition and Behavior, The Netherlands, Radboud University Medical Center, Nijmegen, The Netherlands; 8 Department of Psychiatry, University of Oxford, Oxford, UK; 9 Department of Medicine, Faculty of Medicine and Health Sciences & Institute of Neurosciences, Universitat de Barcelona, and Institut d’Investigacions Biomèdiques August Pi i Sunyer (IDIBAPS), Barcelona, Spain; 10 Clinic and Policlinic for Psychiatry and Psychotherapy, University Medical Center Hamburg-Eppendorf, Hamburg, Germany; 11 Danish Research Centre for Magnetic Resonance (DRCMR), Centre for Functional and Diagnostic Imaging and Research, Copenhagen University Hospital - Amager and Hvidovre, Copenhagen, Denmark; 12 Department of Radiation Sciences, Diagnostic Radiology, Umeå University, Umeå, Sweden; 13 Institute of Sports Medicine Copenhagen (ISMC) and Department of Neurology, Copenhagen University Hospital Bispebjerg, Copenhagen, Denmark; 14 Faculty of Psychology and Educational Sciences, University of Geneva, Geneva, Switzerland; 15 UniDistance Suisse, Brig, Switzerland; 16 Swiss National Centre of Competence in Research LIVES, University of Geneva, Geneva, Switzerland; 17 Welcome Centre for Integrative Neuroimaging, University of Oxford, Oxford, UK; 18 Amsterdam Neuroscience, Department of Psychiatry, Amsterdam UMC, Vrije Universiteit, Amsterdam, The Netherlands; 19 Radiography, Department of Technology, University College Copenhagen, Copenhagen, Denmark; 20 Institute for Clinical Medicine, Faculty of Medical and Health Sciences, University of Copenhagen, Copenhagen, Denmark

**Keywords:** Cognitive ageing, Cognitive neuroscience

## Abstract

Higher general cognitive ability (GCA) is associated with lower risk of neurodegenerative disorders, but neural mechanisms are unknown. GCA could be associated with more cortical tissue, from young age, i.e. *brain reserve,* or less cortical atrophy in adulthood, i.e. *brain maintenance*. Controlling for education, we investigated the relative association of GCA with reserve and maintenance of cortical volume, -area and -thickness through the adult lifespan, using multiple longitudinal cognitively healthy brain imaging cohorts (n = 3327, 7002 MRI scans, baseline age 20–88 years, followed-up for up to 11 years). There were widespread positive relationships between GCA and cortical characteristics (level-level associations). In select regions, higher baseline GCA was associated with less atrophy over time (level-change associations). Relationships remained when controlling for polygenic scores for both GCA and education. Our findings suggest that higher GCA is associated with cortical volumes by both brain reserve and -maintenance mechanisms through the adult lifespan.

## Introduction

Does higher intelligence protect against brain atrophy in aging? Numerous findings motivate this question: General cognitive ability (GCA) is positively associated with brain volume and cortical characteristics at various life stages, including young adulthood and older age^1–5^. GCA is consistently associated with all-cause mortality and health, with higher GCA related to lower risk of diseases and lifestyle factors known to negatively affect brain health^4^. In part, associations are still found after controlling for factors such as educational attainment, suggesting that contemporary GCA in itself is of importance^4^. While higher education has been posited as a protective factor against neurodegenerative changes^6,7^, we recently documented in a large-scale study of multiple cohorts that education is not associated with rates of brain atrophy in aging^8^. A more promising candidate influence on brain aging may thus be GCA independently of education. Whether GCA level is predictive of longitudinal cortical change has primarily been investigated in older cohorts, and with mixed results^9–11^. The relationship of GCA level and cortical changes through the adult lifespan has to our knowledge hitherto not been investigated.

In this context, the lifespan perspective is critical and has implications for understanding functional loss in older age. Several studies indicate that people with higher GCA in young adulthood may be at lower risk of being diagnosed with neurodegenerative disorders in older age^4,12,13^. Recent findings from large datasets point to a relationship between family history of Alzheimer´s Disease (AD) and cognitive performance level four decades before the typical age of onset of AD^14^. However, GCA-AD risk associations have not been consistently observed, and mechanistic factors are poorly understood^15^. Possible explanations include both a brain reserve, i.e. “threshold model”^16^, as well as a brain maintenance^17^ account. The brain reserve model would entail that higher GCA as a trait is related to greater neuroanatomical volumes early in life, young adulthood inclusive, thus delaying the time when people fall below a functional threshold of neural resources in the face of neurodegenerative changes with age. This would happen even if such changes in absolute terms are of similar magnitude across different ability levels, i.e. slopes are parallel, indicating “preserved differentiation”^18^, where initial differences in young are upheld with age^16,19^. The brain maintenance^17^, or “differential preservation”^18^ account would on the other hand predict less brain change in adulthood for people of higher ability, and therefore a smaller risk of cognitive decline and dementia^19^. The brain reserve and maintenance accounts of the relationships between GCA, brain characteristics and clinical risk are not mutually exclusive, but their relative impact through the adult lifespan is unknown. Collectively, the current findings indicate a need to understand whether there is a relationship between GCA as a trait and brain changes, independently of education, over the adult lifespan.

We tested whether GCA predicted brain aging as indexed by cortical volume, area and thickness change measured longitudinally in 7002 MRI scans from several European cohorts covering the adult lifespan in the Lifebrain consortium^20^ and the UK Biobank (UKB)^21,22^ (n = 3327, age range 20–88 years at baseline, maximum scan interval of 11 years, see Online Methods for details). To disentangle possible environmental and genetic influences on the relationship between GCA and brain aging, we controlled for educational attainment in the main analyses, and in a second step for polygenic scores (PGSs) for education and GCA^23,24^. Established PGSs are only moderately predictive of GCA^23^, but in view of evidence that the polygenic signal clusters in genes involved in nervous system development^23^, we did expect such scores to explain part of the intercept effect, with no or weaker effects on brain aging. We expected any effects of GCA on cortical changes to apply to all ages, but in view of recent findings of greater relationships between brain and cognitive function in older than younger individuals^3^, we also tested the age interaction. Based on previous findings, including from broader cross-sectional Lifebrain cohorts^25^, and mixed results from smaller longitudinal older cohorts^9–11^, we hypothesized that GCA would be positively related to anatomically widely distributed cortical characteristics through the adult lifespan (intercept effect), but that associations with differences in cortical aging trajectories (slope effects) may be observed to a lesser extent. We expected effects of GCA to be at least partially independent of education^8^, both for intercept and slope associations.

## Results

The main models of associations of GCA with cortical characteristics, and their change, were run separately for samples within the Lifebrain consortium (n = 1129, 2606 scans)^20^ and the UK Biobank (UKB, n = 2198, 4396 scans)^21,22^, and then meta-analyses were run on the results, using the metafor package^26^. Using the estimate and standard error at each vertex, random effects meta-analyses were conducted at each vertex separately. In all main models, sex, baseline age, scanner, time (interval from baseline) and education were entered as covariates. In modeling the effects of GCA on cortical characteristics (*level-level analyses*), GCA was entered as the predictor (explanatory variable), whereas in modeling the effects of GCA on brain aging (*level-change analyses*), the interaction term of GCA × time was entered as the predictor, and education × time was entered as an additional covariate along with GCA and education. Since brain aging (i.e. change) was of chief interest, we did not include intracranial volume (ICV), which is stable, in the main analyses. For direct comparison, we also then present level-level analyses without controlling for ICV. This was also chosen given the paucity of evidence for region-specific associations, and previous studies indicating that neuroanatomical volume in and of itself, when controlling for sex, may be associated with GCA^9,25^. Results from models including ICV, as well as models without education, as covariates, can be found in the Supplementary Information (SI). Additional analyses included the interaction term baseline age × time as a covariate, and in one set of analyses we entered the interaction term baseline age × time × GCA as predictor (with relevant two-way interaction terms as covariates), to test if effects differ reliably across the lifespan.

### GCA level: brain level analyses

**Figure 1:**
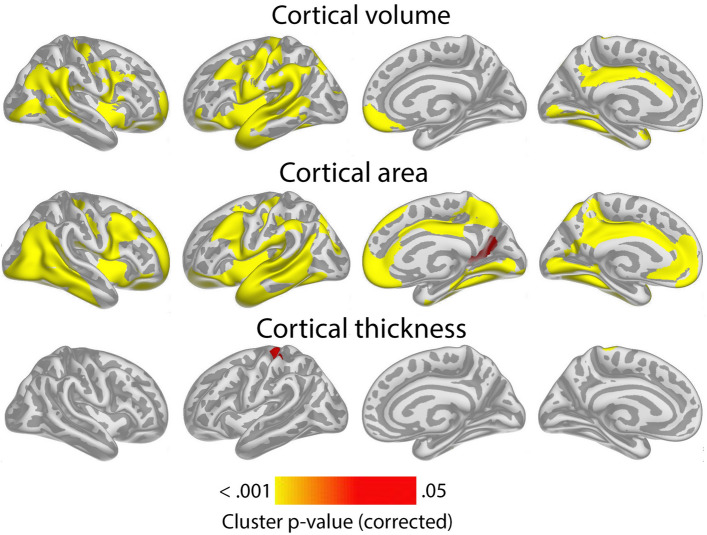
Level-level associations: meta-analytic cluster p-value maps of the associations of general cognitive ability (GCA) and cortical characteristics, controlled for education. Meta-analytic cluster p-value maps (Lifebrain and UKB) of the relationships between GCA at baseline and cortical characteristics are shown, when age at baseline, sex, time (since first scan) and education are controlled for (p < 0.05, corrected using a cluster-forming p-value threshold of p < 0.01). Relationships are shown, from left to right for each panel: right and left lateral view, right and left medial view.

Cluster p-value maps across Lifebrain and UKB for the relationship of GCA and cortical characteristics controlled for education, are shown in Fig. 1. For cortical volume and area, there were widespread positive associations of GCA bilaterally across the cortical mantle seen in all lobes. For area, significant effects were seen across 47.6% and 44.2% of the left and right hemisphere surface, respectively. For volume, similar numbers were 37.4% and 19.5% for left and right, respectively.

For cortical thickness, only minor positive effects were seen, in proximity of the left central sulcus, covering only 1.1% of the surface.

Results of analyses per sample, controlling and not controlling for education are shown in Supplementary Figs. 1 and 2. Effects were largely similar, though slightly more restricted spatially, when controlling, than when not controlling for education. When adding ICV as a covariate, the intercept effects for cortical volume and area in the meta-analysis shown in Fig. 1 became non-significant, with only a very small effect on cortical thickness in the left hemisphere remaining (see Supplementary Fig. 3), pointing to these being broad effects grounded in greater neuroanatomical structures in general, rather than being region-specific.

To show effect sizes, we calculated the effect of 1 SD increase in GCA on cortical volume. Across Lifebrain and UKB, 1 SD higher GCA was associated with 1.0% larger cortical volume. Effect size maps for level-level analyses showing the regional variation in effect sizes for each sample separately are shown in Supplementary Fig. 4. Effect sizes were numerically smaller in Lifebrain (0.6%) than in UKB (1.3%). Restricting the analyses to regions where significant effects were seen, 1 SD increase in GCA was associated with 2.0% larger cortical volume in Lifebrain and 1.6% larger volume in UKB, but please note that these latter effect sizes are inflated by being within significant regions. Similar analyses for cortical area showed that 0.8% larger area was associated with 1 SD higher GCA across the cortex, with effects being 0.6% in Lifebrain and 0.9% in UKB. Restricting the analyses to regions where significant effects were seen, 1 SD increase in GCA was associated with 1.6% larger cortical volume in Lifebrain and 1.2% larger volume in UKB, with the same caveat as above. For thickness, effects were minute: 0.06% across studies (Lifebrain 0.04%; UKB 0.08%). Within significant clusters (UKB only), the effects of 1 SD higher GCA was 0.8%.

### GCA level: brain change analyses

**Figure 2:**
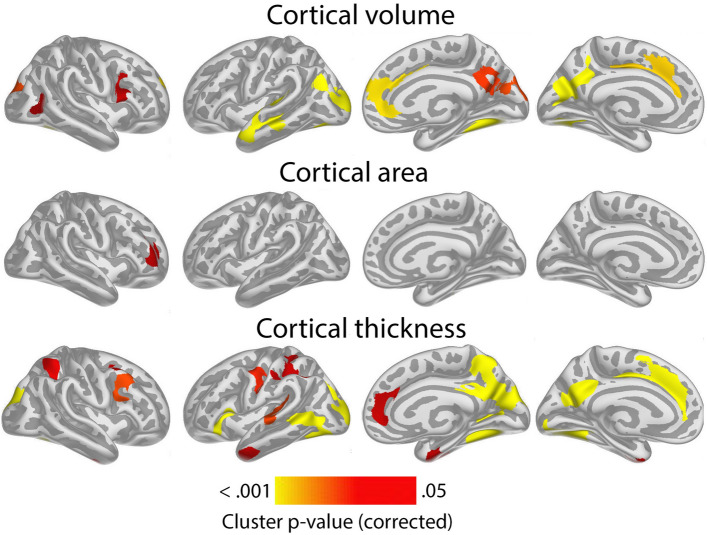
Level-change associations: meta-analytic cluster p-value maps of the associations of general cognitive ability (GCA) with change in cortical characteristics, controlled for the effect of education. The association is shown for the interaction of GCA at baseline and time (interval since baseline scan), when age, sex, time, GCA, education, and the interaction of education and time, are controlled for (p < 0.05, corrected using a cluster-forming p-value threshold of p < 0.01). Significant regions are shown, from left to right for each panel: right and left lateral view, right and left medial view.

Having confirmed the expected positive relationships between GCA and cortical volume and area controlled for education in terms of an intercept effect, we investigated the question of slope effects. Associations of GCA level at baseline and change in cortical characteristics, controlled for education, are shown in Fig. 2. As expected, effects were more spatially limited than those seen for intercept models, with only restricted regions showing significant relationships: Higher baseline GCA was associated with less regional cortical volume reduction in the left middle cingulate gyrus, a medial area around the central sulcus and a part of the lingual gyrus. The most extensive effects were seen for thickness change, where higher baseline GCA was associated with less thinning in regions corresponding to the volume effects, in addition to parts of the right anterior and lateral temporal cortex and an area in the most medial part of the intersection between the central sulcus and the superior frontal cortex. No associations with area change were observed Taken together, this means that the observed positive associations of GCA with volume change primarily reflect less cortical thinning with higher GCA. (See Supplementary Fig. 5 for result for each subsample separately).

Associations of GCA with cortical change were essentially unaffected by adding ICV as a covariate (Supplementary Fig. 6).

**Figure 3:**
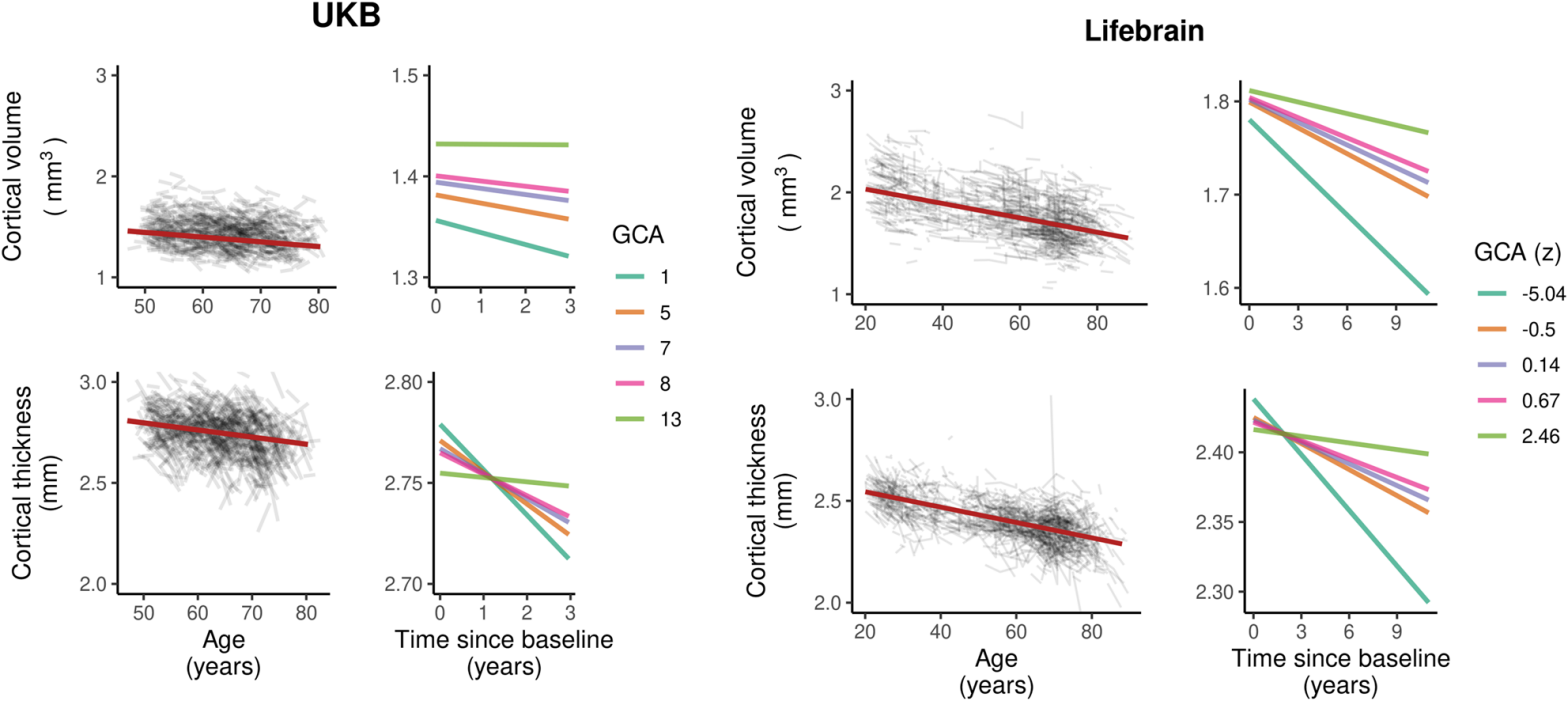
Cortical change trajectories according to general cognitive ability (GCA). Trajectories are shown per quintile of GCA for illustrative purposes, for mean cortical volume and thickness change in the analysis model and regional significant sites of associations in each cohort (shown in Supplementary Fig. 5). For UKB, the quintiles refer to actual scores from min to max (0–13) on the test, whereas for Lifebrain, the quintiles refer to z-scores (where mean is zero) min to max for the samples.

In order to illustrate the GCA-cortical change relationships, and to characterize consistency of effects across samples (UKB and Lifebrain), we plotted the generalized additive mixed model (GAMM) for the different GCA quintiles, from lowest to highest (Fig. 3), depicting change trajectories for average cortical volume and thickness within the regions showing significant GCA x time associations. Across samples, subgroups with higher GCA started with higher volume and had less volume loss over time. For instance, on average, people with maximum cognitive score in UKB are expected start out with a regional average cortical volume of 1.72 mm^3^ that would be maintained for the next three years, whereas those with the lowest GCA would on average start out with 1.64 mm^3^ and decrease to 1.61 mm^3^ over the next three years. Thus, the greatest GCA-associated differences in cortical volume are found in the intercepts (level), whereas differences in slope (change) are smaller in the follow-up period. For cortical thickness, the change trajectories were also very consistently ordered, but those with higher GCA did not uniformly have thicker cortex at first timepoint in these areas. Rather, differential rates of cortical thinning over time were critical in creating cortical thickness differences in these regions in aging. This was evident in both samples, but especially pronounced in UKB.

**Figure 4:**
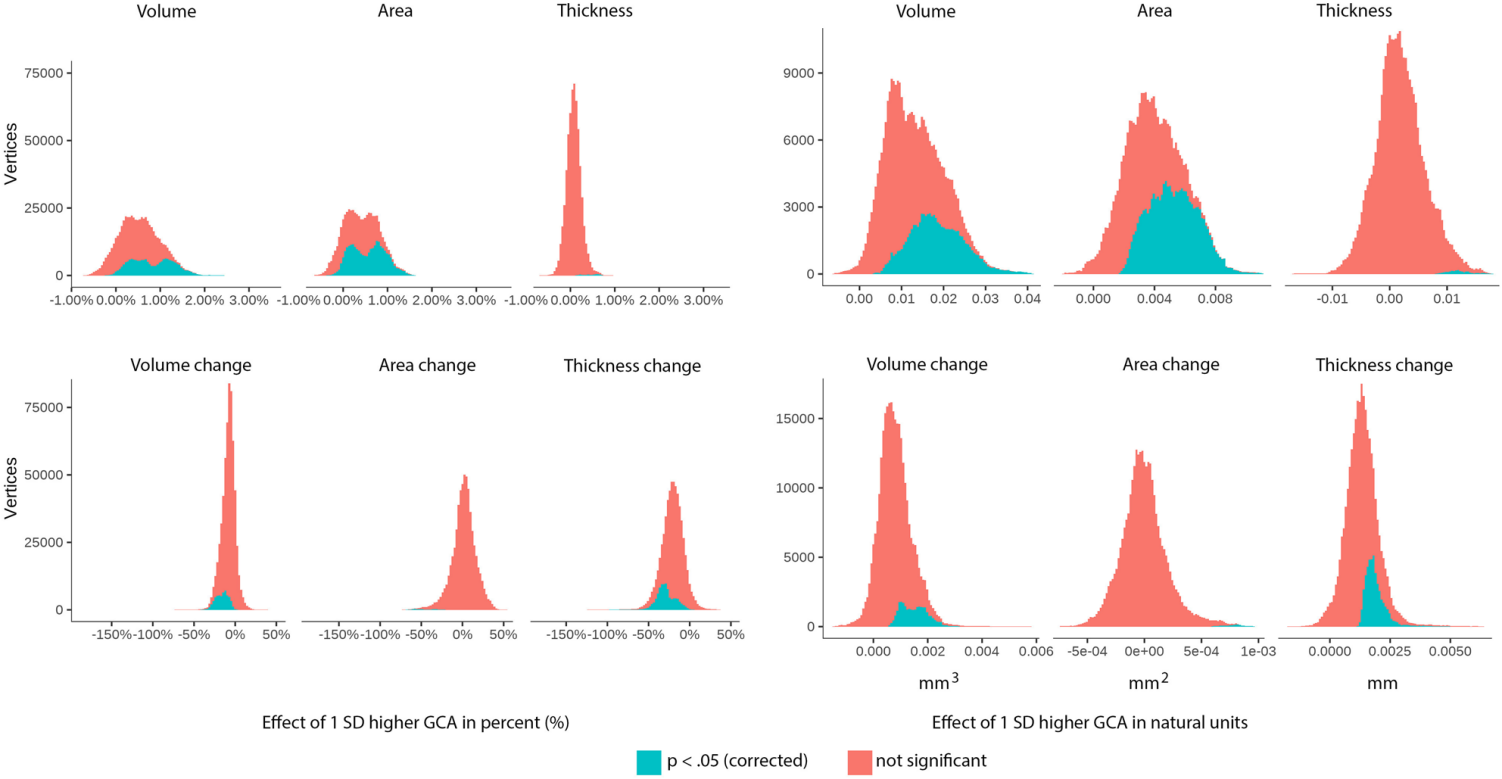
Cortical vertex-wise distributions of effects of 1 SD higher GCA in percent and natural units. Upper panel: effect distributions for level-level associations. Lower panel: effect distributions for level-change associations.

To further assess effect sizes, we created histograms of the vertex-wise distribution of effects of one SD higher GCA for each metric for absolute volume, area and thickness as well as their change, shown in Fig. 4. (For cortical distributions of such effect sizes per sample, see Supplementary Fig. 7). As can be seen, almost all vertices show positive level–level relationships between GCA and volume and area. For thickness, the distribution is only slightly shifted to the right of zero, confirming the weak GCA-thickness relationships. As for GCA level-brain change, the histograms showed that for area, effects were distributed almost perfectly around zero. For volume, there was a clear shift rightwards, meaning that higher GCA tended to be related to less volume reductions, but substantially less than for the offset effects. Cortical thickness showed the most rightward skewness of the distribution, much larger than for the offset results. Inspecting all histograms, it is clear that higher GCA is related to larger cortical volume and area, and less thickness change.

### Influence of polygenic scores (PGSs) for GCA and education on the level-level and level-change associations

**Figure 5:**
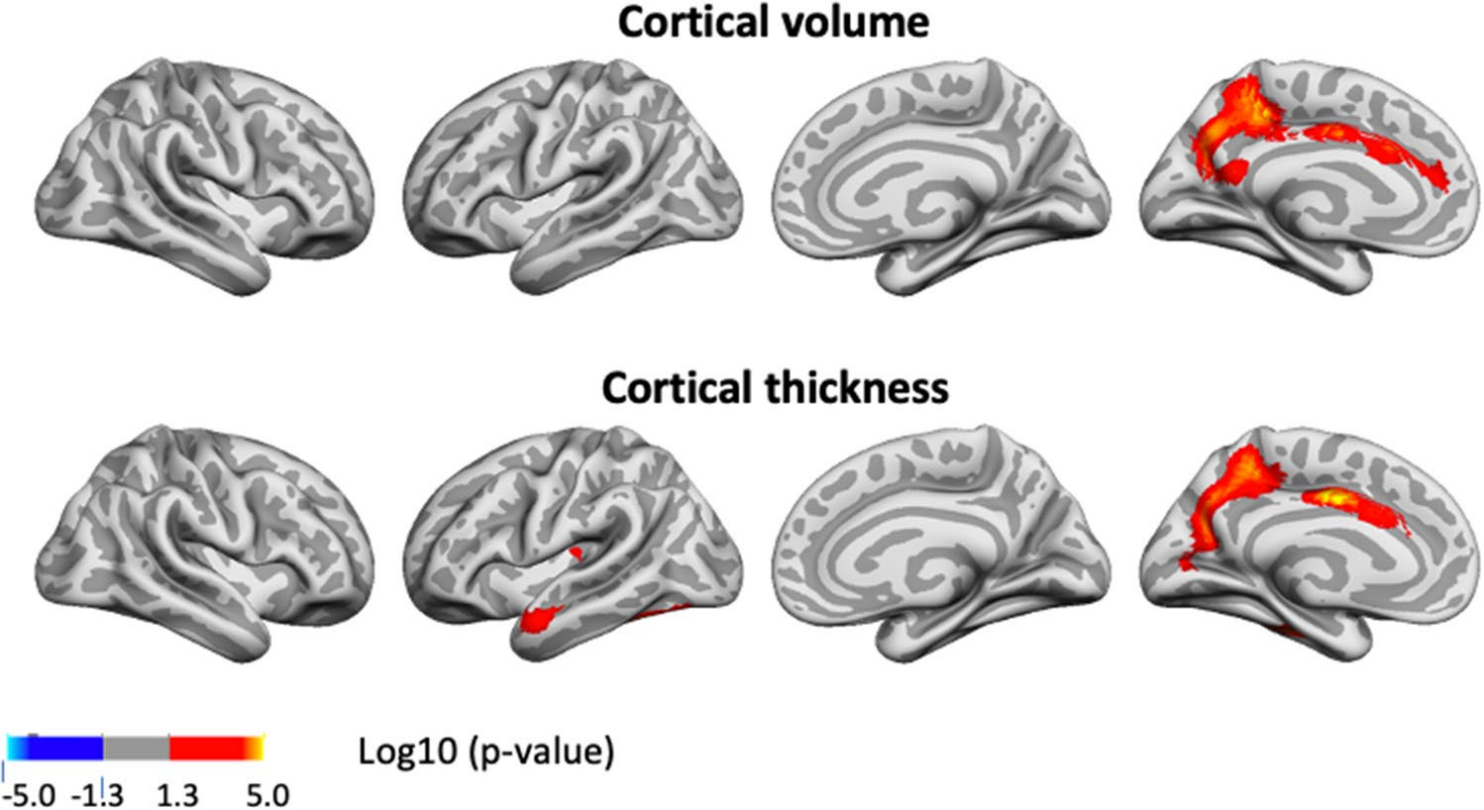
P-value maps of associations of general cognitive ability (GCA) and change in cortical characteristics in the UKB, controlled for the effect of education and polygenic scores (PGSs) for education and GCA over time. The significant regions are shown for the interaction of GCA at baseline (with genetic ancestry factors regressed out) and time (interval from baseline scan), when age, sex, time, GCA, education, and the interactions of education by time, and PGSs by time, are controlled for (p < 0.01, corrected using a cluster-forming threshold of p < 0.01). Regions are shown, from left to right for each panel: right and left lateral view, right and left medial view. No significant regions were seen for cortical area.

Next, we investigated whether effects were maintained when covarying for established PGSs for GCA and education in the UKB^23,24^. Fifty-two participants in the main models were excluded due to missing genetic data. In these analyses, we regressed out the first ten genetic ancestry factors (GAFs) from the GCA variable prior to analysis. The intercept associations of GCA and cortical characteristics that were observed in the main model (Fig. 1) largely remained when controlling for the PGSs, but the extent of the significant regions were somewhat reduced for cortical volume and area (Supplementary Fig. 8). The associations of GCA and cortical change largely remained and were only slightly attenuated when controlling for PGSs for GCA and education (Fig. 5; compare to UKB results in Supplementary Fig. 5).

### Influence of age on the level-change associations

**Figure 6:**
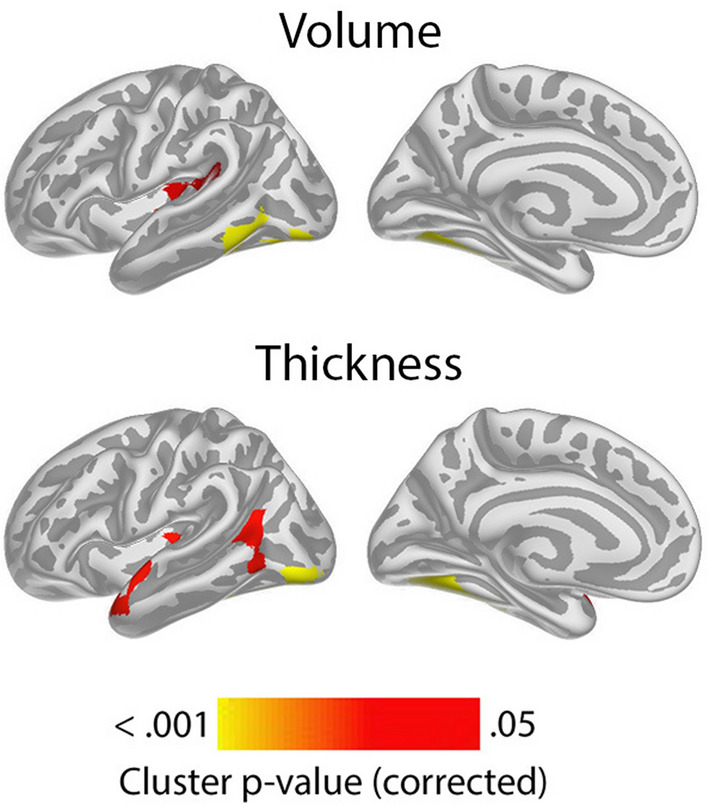
Meta-analytic cluster p-value maps for interactions of level general cognitive ability (GCA), by baseline age by time on cortical volume and thickness. The significant regions are shown for the interaction of GCA at baseline, by age at baseline by time (interval since baseline scan), when age, sex, time, GCA, education, and the interactions education by time, GCA by time, and baseline age by time, are controlled for (p < 0.05, corrected using a cluster-forming p-value threshold of p < 0.01). Significant regions are shown, for each panel for left lateral and medial view. No significant regions were seen for cortical area, or for the right hemisphere.

We next tested the three-way interaction baseline GCA × baseline age × time, to see whether the level-change associations differed reliably across the lifespan. Significant interaction effects were seen for change in small regions of the left hemisphere, mostly laterally for volume, and for slightly more extended regions, for cortical thickness (see Fig. 6).

**Figure 7:**
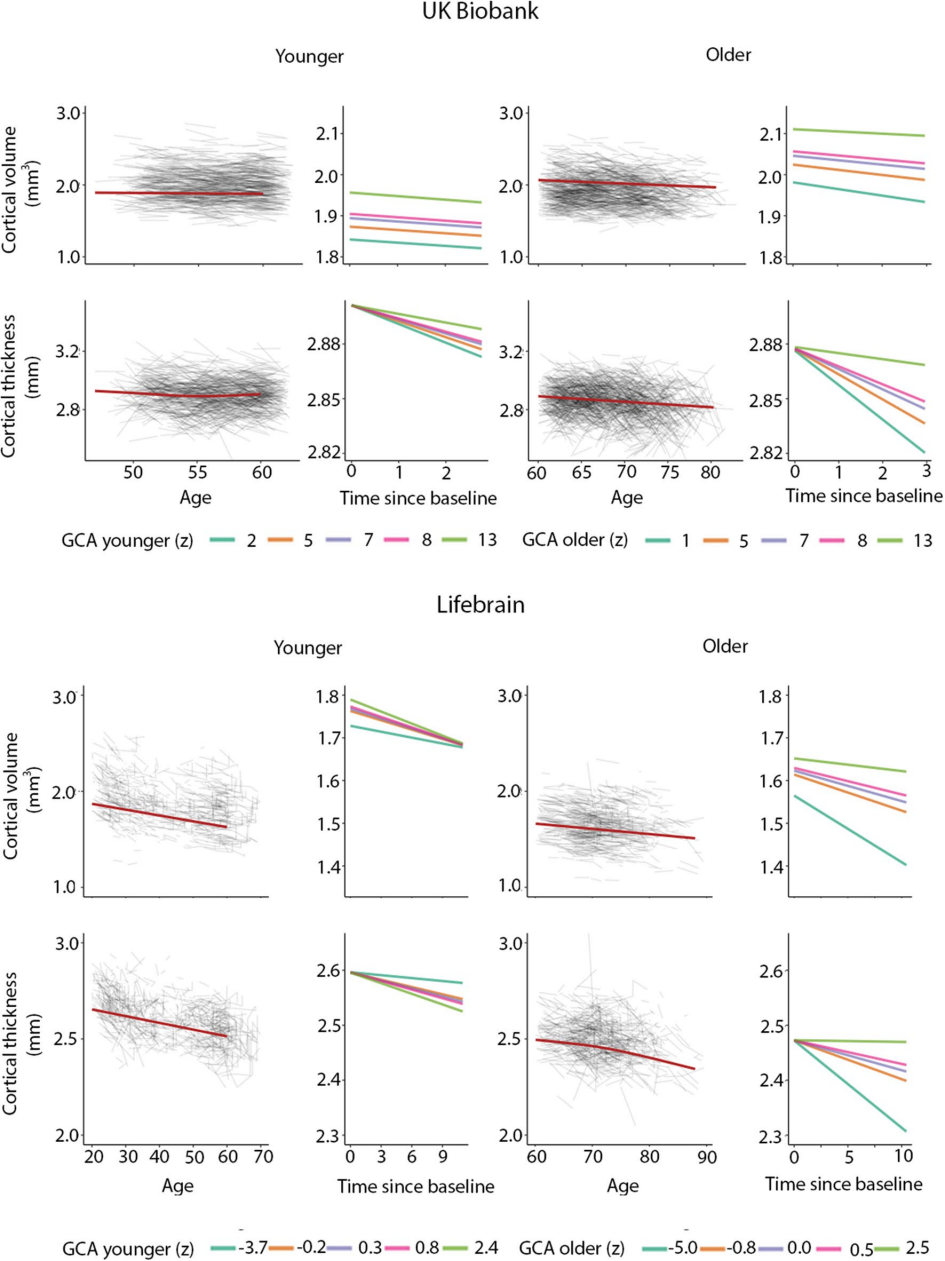
Cortical volume and thickness change trajectories according to general cognitive ability (GCA) for young and older adults. For illustrative purposes, expected trajectories are shown for young (< 60 years at baseline) and older (> 60 years at baseline) in UKB (top panels) and Lifebrain (lower panels) per quintile of GCA, for mean cortical volume and thickness in the analysis model and regional significant sites shown in Fig. 6. For UKB, the quintiles refer to actual scores from min to max (0–13) on the test, whereas for Lifebrain, the quintiles refer to z-scores (where mean is zero) min to max for the samples.

The positive three-way interactions of baseline GCA × baseline age × time indicates that higher level of GCA is associated with less atrophy at distinct ages. To visualize these interaction effects, we divided the cohorts according to whether participants were above or below age 60 years. This division point was chosen in view of it being an approximate age at which select cognitive and regional cortical volume and thickness changes have been reported to accelerate in longitudinal studies^27,28^. In order to explore how GCA level related to cortical thickness change over time across the two age groups, we plotted the expected cortical change trajectories, within the significant regions shown in Fig. 6, as a function of GCA, with each sample divided into quintiles, from lowest to highest GCA. The plots are shown in Fig. 7. While GCA level was weakly, and in Lifebrain even inversely related to atrophy in these regions in the younger group, the expected trajectories for the older group were relatively consistently ordered so that persons with a higher GCA level had less decline, especially of cortical thickness. The GCA quintile differences are more pronounced in the older group, suggesting the latter half of the lifespan is driving the interaction. As one outlier in the older group in Lifebrain was noted as having a high cortical thickness value for age at the first timepoint in the region of interest, we carefully checked this segmentation, but found no sign of flawed segmentation, and thus decided to keep this person in analyses.

## Discussion

The current study provides novel findings on GCA not only as a marker of brain characteristics, but also of brain changes in healthy aging. The finding that higher GCA level is associated with larger neuroanatomical structures to begin with, i.e. greater brain reserve, confirms findings in previous studies of various age groups^1–4,25,29^. While level of GCA has been associated with cortical change in some older groups^10,11^, but not others^9^, the current demonstration of an association of GCA levels, controlled for education, on cortical volume and -thickness declines through the lifespan in multiple cohorts across a relatively long follow-up time, constitutes a novel finding. Also, the finding of an age-interaction with pronounced effects of GCA on cortical thinning and volume changes only in older ages in select regions, is novel.

The association of GCA level and cortical change appears relatively moderate. This may explain why such associations have not previously been consistently found. The “effect” of GCA on cortical change must be viewed in relation to the intercept effects, which, as shown here, constitute a major source of GCA-related cortical volume variation through the lifespan: Those with higher GCA have greater cortical area to begin with, yielding higher cortical volumes in young adulthood. We have previously found that cortical area seems in part determined neuro-developmentally early on, is associated with GCA, and shows parallel trajectories for higher and lower GCA groups^1^. As there is a relatively minor age change in area, compared to thickness^28^, slope effects on cortical volume are chiefly caused by moderately different rates of cortical thinning for people of differential cognitive ability. Differences in cortical thinning are thus key to the maintenance effects of GCA, whereas early differences in cortical area drive the intercept effect. Through the adult lifespan, both will affect cortical volume.

It is of interest that these GCA-brain change associations were found when education was controlled for, suggesting that the contemporary GCA level may not only be related to brain reserve^16^ to begin with, and preserved differentiation^18^, but also brain maintenance^17^, and differential preservation^18^. This is evident from the—across samples—consistently steeper slopes of regional cortical decline with lower GCA (as illustrated in Fig. 3). With our recent findings on the variable nature of education-brain-cognition relationships, as well as education not being associated with atrophy rates in aging^25,30^, this points to the component of GCA not being associated with education variance as a more promising candidate for predictive or potentially protective effects on brain aging. There is evidence that education may serve to increase GCA^31,32^. However, while GCA level may be impacted, slope, i.e. cognitive decline, is likely not^32,33^. There is also evidence to suggest that education, without mediation through adult socioeconomic position, cannot be considered a modifiable risk factor for dementia^34^.

While one would then think the underlying mechanism in the observed GCA-brain change relationships may be genetic, known genetic factors only partially explained relationships, as effects remained after controlling for PGSs for general cognitive ability and education. However, the PGSs are known to be only moderately predictive of GCA^23^, and genetic pleiotropic effects on GCA and cortical characteristics and their change may still likely be part of the underlying mechanism. While it has been suggested that GCA may associate with differences in epigenetic age acceleration, it was recently reported that such epigenetic markers did not show associations with longitudinal phenotypic health change^35^. While it is possible that individual differences in epigenetic age acceleration in older age could be caused by e.g. behaviors associated with intelligence differences over the life course, differences in epigenetic markers and GCA could also both be the result of a shared genetic architecture or some early, including in-utero, environmental event^35,36^.

A significant three-way interaction of baseline GCA by baseline age by time on regional cortical thickness changes was observed by meta-analysis across cohorts. These effects indicated that higher level of GCA is more associated with less atrophy at older ages. However, as these regional interaction effects were highly restricted, and also seemed to rest in part on unexpected, albeit weakly, inverse direction of smaller effects in younger^28^ age in Lifebrain, we consider them tentative until replicated. The higher baseline age of the UKB sample, also may make it less suitable to study adult lifespan interactions. Moreover, greater power would be desired to study three-way interactions of possibly smaller effect size.

Some further limitations to the present study should also be noted: The samples included are heterogeneous and may have varying degrees of representativeness of the populations of origin, indeed, lack of population representativity is known^37^. Data from relatively short time periods were used. Changing exposure trends over time, in health, education, and welfare, may thus relate to age at baseline, and could have effects that could not readily be studied in isolation here. Furthermore, as cognitively healthy participants were recruited, sample representativity may vary with age. Since persons with known neurodegenerative disorders were excluded, results cannot readily be generalized to persons suffering from various types of dementia. To shed light on the potential genetic contribution to the observed GCA-cortical change relationships, we controlled for PGSs for GCA and educational attainment. While these results indicated negligible genetic contributions, direct investigation of the genetic relations using standard methods, e.g. linkage disequilibrium score regression^38^, may be better suited to investigate this, when large-scale GWAS for longitudinal cortical changes in adulthood becomes available. Finally, change-change relationships between GCA and cortical characteristics could not readily be addressed in the present samples with similar models, due to variability in availability of comparable test data across timepoints. In a lifespan perspective, we know that such relationships do exist, in that both brain and cognition increase in development and decline in aging^17,27,28,39^. However, to what extent individual differences in GCA *change* are related to individual differences in cortical trajectories in the present samples, is beyond the scope of this study.

In conclusion, the present study shows that with higher GCA, primarily brain reserve, but also brain maintenance yield higher cortical volumes through the adult lifespan. These effects were seen when controlling for effects of education. As there is otherwise scarce evidence so far that human behavioral traits are associated with differential brain aging trajectories, this is of great interest to investigate further. While controlling for known polygenetic markers for GCA and education did not substantially diminish the effects, the underlying mechanisms may still be related to genetic pleiotropy. However, this leaves open the possibility that factors associated with increased GCA other than education, and possibly genes, could serve to diminish cortical atrophy in aging. Such factors affecting normal individual differences in GCA are not known with certainty, but as childhood GCA is highly predictive of GCA in aging^40^, they likely work at developmental, rather than adult and senescent stages.

## Materials and methods

The UK Biobank (UKB)^22^ and the Lifebrain samples are described in Table 1. The samples from the European Lifebrain (LB) project (http://www.lifebrain.uio.no/)^20^ included participants from major European brain studies: the Berlin Study of Aging II (BASE II)^41^, the BETULA project^27^, the Cambridge Centre for Ageing and Neuroscience study (Cam-CAN)^42^, Center for Lifebrain Changes in Brain and Cognition longitudinal studies (LCBC)^1^, and the University of Barcelona brain studies (UB)^43–45^.

**Table 1:** Overview of sample characteristics of included cohorts.

Sample	Lifebrain			UKB		
N participants	1129			2198		
N Females	576			1136		
N scans	2606			4396		

	M	SD	Range	M	SD	Range
Baseline age	55.5	18.4	20.0–88.0	20.0	7.1	47.0–80.3
Scan interval	3.7	2.7	0.2–11.0	2.3	0.1	2.0–3.0
Education	14.9	3.1	2.0–20.0	14.0	2.3	10.0–16.0
GCA	0.0	1.0	− 5.0–2.5	6.8	2.0	1.0–13.0

Age, education, and scans intervals (since baseline) are given in years. General Cognitive Ability (GCA) for Lifebrain is standardized per sample for first timepoint. GCA is given in z-scores for Lifebrain (see Supplementary Methods), and test-scores for UKB. *M* Mean, *SD* Standard Deviation.

GCA was measured by partially different tests in the different cohorts. National versions of a series of batteries and tests were used, see SM for details. These included the UKB Fluid Intelligence test^46^, tests from the Wechsler batteries^47–49^ combined with the National Adult Reading Test (NART)^50^, the Cattell Culture Fair Test^51^ combined with the Spot The Word task^52^, as well as local batteries, for which procedures are described in SM and elsewhere^53,54^. It is clearly a limitation that content and reliability of the GCA measures may vary, but there is reason to assume that the measures index partally similar abilities. For instance, the UKB fluid intelligence measure has been shown to have moderate to high reliability, and correlated > 0.50 with a measure of GCA created using 11 reference tests, including NART and Wechsler measures^55^. See SM for further details.

MRIs were processed using FreeSurfer, version 7.1 for Lifebrain*,* and version 6.0 for UKB (https://surfer.nmr.mgh.harvard.edu/https://surfer.nmr.mgh.harvard.edu)^56–59^. We ran vertex-wise analyses to assess regional variation in the relationships between cortical structure and the measures of interest, i.e. GCA and the interaction of GCA × time. Cortical surfaces were reconstructed from the same T1-weighted anatomical MRIs, yielding maps of cortical area, thickness and volume. Surfaces were smoothed with a Gaussian kernel of 15 mm full-width at half-maximum. Spatiotemporal linear mixed models^60,61^ were performed running on MATLAB R2017a (using FreeSurfers ST-LME package https://surfer.nmr.mgh.harvard.edu/fswiki/LinearMixedEffectsModels), for each of the samples separately, with GCA, and then additionally with the interaction term of GCA and time in turn as predictors, and sex, baseline age, scanner, time (interval since baseline scan) and education were entered as covariates unless otherwise noted. These models also account for the spatial correlation between residuals at neighboring vertices and the temporal correlation of residuals within repeated measurements of single participants. Surface results were tested against an empirical null distribution of maximum cluster size across 10 000 iterations using Z Monte Carlo simulations, yielding results corrected for multiple comparisons across space (p < 0.01 corrected)^62^.

All studies were conducted, and all methods performed, in accordance with relevant guidelines and regulations as set forth by the relevant authorities, including the Declaration of Helsinki, all participants gave informed consent, and subprojects were approved by the relevant ethical review boards. UK Biobank has approval from the North West Multi-centre Research Ethics Committee as a Research Tissue Bank approval. The Lifebrain project was approved by Regional Committees for Medical Research Ethics–South East Norway. For additional details, see SM. Screening criteria were not identical across studies, but participants were recruited to be cognitively healthy and did not suffer from neurological conditions known to affect brain function, such as dementia. All samples consisted of community-dwelling participants, some were convenience samples, whereas others were contacted on the basis of population registry information. Further details on samples, GCA measures, MRI acquisition and processing and statistical analyses, are presented in SM. The Lifebrain data supporting the results of the current study are available from the PI of each sub-study on request (see SM), given approvals. UK Biobank data requests can be submitted to http://www.ukbiobank.ac.uk. Computer code used for the analyses is available on github: https://github.com/Lifebrain/p032-gca-brain-change.

## Supplementary Information

Supplementary Information.
